# Aqueous Synthesis of Strontium Ruthenate(VI) Oxyhydroxides and Their Crystal Structure Solution from Microcrystals

**DOI:** 10.1021/acs.inorgchem.5c03066

**Published:** 2025-09-01

**Authors:** Mark Crossman, Craig I. Hiley, Helen Y. Playford, Ronald I. Smith, Thomas C. Hansen, Jeremiah P. Tidey, Richard I. Walton

**Affiliations:** † Department of Chemistry, 2707 University of Warwick, Gibbet Hill Road, Coventry CV4 7AL, U.K.; ‡ ISIS Neutron and Muon Facility, Rutherford Appleton Laboratory, Didcot OX11 0QX, U.K.; § Institut Laue-Langevin, 71 Avenue des Martyrs CS 20156, Grenoble Cedex 9 38042, France; ∥ Department of Physics, University of Warwick, Gibbet Hill Road, Coventry CV4 7AL, U.K.

## Abstract

Investigation of the earliest stages of hydrothermal reactions between KRuO_4_ and either Sr­(NO_3_)_2_ or SrO_2_ allows the isolation of phase-pure SrRuO_3_(OH)_2_, a hitherto uncharacterized Ru­(VI) oxyhydroxide, previously mentioned in the literature as SrRuO_4_·H_2_O. Further exploration of synthesis conditions with excess of strontium salt yields a second Ru­(VI) phase, Sr_3_Ru_2_O_8_(OH)_2_ at 200 °C. Both structures were solved by 3D-electron diffraction and refined using powder neutron diffraction (SrRuO_3_(OH)_2_: monoclinic *P*2_1_/*n*, *a* = 9.9903(3) Å, *b* = 7.7023(2) Å, *c* = 17.3677(6) Å, β = 89.353(2)°; Sr_3_Ru_2_O_8_(OH)_2_: tetragonal *P*4̅, *a* = 13.2206(5) Å *c* = 5.4852(2) Å). The two Ru­(VI) materials each contain isolated trigonal bipyramidal ruthenium centers with axially positioned hydroxides with longer Ru–OH bond distances than the equatorial Ru–O distances. Infrared spectroscopy corroborates the presence of hydroxide, with O–H stretching bands that are red-shifted upon deuteration. X-ray absorption near-edge spectroscopy at the Ru K-edge confirms the +6 oxidation state of Ru, and magnetization measurements show two unpaired electrons per Ru associated with the 4d^2^ electronic configuration in both materials. The crystal structures, particularly the local environment of Ru, are compared with other reported Ru­(VI) compounds, and derived bond valence sum parameters give consistent results for ruthenate­(VI) oxides and oxyhydroxides.

## Graphical abstract

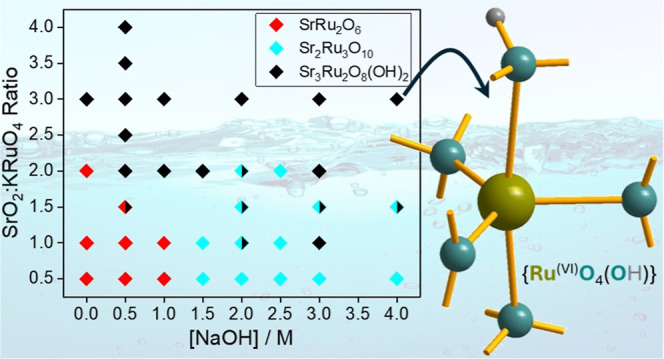

## Introduction

A wide variety of alkali-earth ruthenates have been reported that show a striking diversity of crystal structures, electronic properties, and magnetic behaviors.^1,2^ The case of strontium ruthenates is highly illustrative, with itinerant ferromagnetism in the perovskite SrRuO_3_,^3^ unconventional superconductivity observed in the *n* = 1 Ruddlesden–Popper Sr_2_RuO_4_,^4–6^ metamagnetism in the “strange metal” Sr_3_Ru_2_O_7_ that has an *n* = 2 Ruddlesden–Popper structure,^7,8^ and unusually high-temperature antiferromagnetic order in the layered honeycomb structure of SrRu_2_O_6_.^9^ These materials contain either Ru^4+^ or Ru^5+^, and traditionally they have been prepared by the conventional high-temperature approaches of solid-state chemistry. In more recent years, the use of hydrothermal conditions has yielded a number of alkali-earth (Ca, Sr, and Ba) ruthenates,^10,11^ including the aforementioned SrRu_2_O_6_,^9^ which apparently is not accessible by other synthetic routes, an analogue BaRu_2_O_6_ with a related layered honeycomb structure and similar magnetic behavior,^12^ and the hexagonal 8H-perovskite Ba_4_Ru_3_O_10.2_(OH)_1.8_, with a complex structure containing partially occupied Ru sites showing regions of ordering on the nanoscale.^13^ In the strontium ruthenate system, Marchandier et al. also discovered the compositions Sr_2_Ru_3_O_9_(OH) and Sr_2_Ru_3_O_10_ by increasing pH from the neutral conditions used to form SrRu_2_O_6_, as well as three new barium ruthenate phases.^11^

The recent reports of hydrothermal synthesis of ruthenates have used very mild conditions, typically around 200 °C, and have made use of the Ru­(VII) precursor KRuO_4_ as a convenient and reactive reagent that cleanly yields the alkali-earth ruthenates in a single step, from solutions of alkali-earth salts, with adjustment of pH allowing phase selectivity.^10,11^ It is notable that the materials accessed by this route usually contain Ru­(V), which suggests the use of a high oxidation state ruthenium precursor as a general approach to the preparation of ruthenium oxides in high oxidation states, aided by the partner electropositive alkali earths that stabilize high oxidation states of transition metals by an inductive effect.^14^ Ru­(VI) has also been found in a number of oxides and oxyhydroxides. Most of these have so far generally been prepared in solid-state reactions typically using a peroxide as a reagent, such as for Cs_2_RuO_4_,^15^ or CsK_5_Ru_2_O_9_,^16^ and in some case also under oxygen pressure such as for Na_2_RuO_4_,^17^ while unusually, the material CuRuO_2_(OH)_4_ was precipitated from KRuO_4_ and aqueous Cu^2+^.^18^

Given the exotic magnetic and electronic properties of alkali-earth ruthenates that continue to attract attention, and their rich chemistry that clearly offers scope for further discovery of materials, it would be of benefit to understand the crystallization of the materials with a greater level of detail. We herein report a study of crystallization of strontium ruthenates from aqueous KRuO_4_ where we explore mild conditions and high Sr/Ru ratios. This leads to the isolation of two strontium ruthenate (VI) oxyhydroxides, phases never previously structurally characterized. We discuss the structures in comparison to other known Ru­(VI) oxides and oxyhydroxides and examine trends in the structure and bonding in these materials.

## Experimental Section

Hydrothermal crystallization was carried out using ∼20 mL Teflon-lined, stainless-steel autoclaves. The chemical precursors were used as supplied: KRuO_4_ (STREM Chemicals, 98%), SrO_2_ (Sigma-Aldrich), and Sr­(NO_3_)_2_ (Sigma-Aldrich). Either deionized water was used as reaction medium or D_2_O (Apollo Scientific, 99.9%), in preparation for the neutron diffraction experiments, and in some cases 0.5 M NaOH when pH was varied (see Results and Discussion section for specific synthetic details). After heating the reagent mixture for chosen temperature and time, the autoclave was allowed to cool naturally, and the solid product was recovered by Büchner filtration. During filtration, the material was washed with deionized water and finally acetone to dry the product prior to analysis.

Initial sample assessment and phase identification were carried out using a Siemens D5000 diffractometer with Cu Kα radiation (Kα_1_ λ = 1.54056 Å and Kα_2_ λ = 1.54443 Å). Diffraction angles of 5–75° were scanned with a step size of 0.02° and a time per step of 1 s. Alternatively, a third-generation Malvern Panalytical Empyrean instrument was used with multicore (iCore/dCore) optics and a Pixcel3D detector operating in a 1D receiving slit mode: here, Cu Kα radiation was used and diffraction angles of 5–75° 2θ were scanned with a step size of 0.0131° and 2.1 s per step. In situ X-ray thermodiffractometry experiments were performed by using a Bruker D8 ADVANCE diffractometer with Cu Kα radiation and a VÅNTEC-1 high-speed detector. The sample housing was an Anton Paar XRK 900 reaction chamber controlled by a TCU 750 temperature unit. Sample heating was carried out at 0.2 °C per second from 30 °C up to 900 °C, with diffraction patterns measured at 15 °C intervals temperature while temperature was held constant. Data were recorded over diffraction angles 8–60° 2θ with a step size of 0.0163° and a time per step of 1.2 s. Synchrotron powder XRD data were collected using the I11 beamline at Diamond Light source, UK.^19^ The X-ray wavelength was determined using a Si standard as being 0.825960(1) Å, and data were collected using a PSD detector from samples contained in 0.2 mm borosilicate capillaries. Samples were spun during data collection to improve powder averaging.

Infrared (IR) spectroscopy was carried out on a Bruker ALPHA II FT-IR spectrometer on solid powders over a range of 4000 to 450 cm^–1^ and corrected using a background scan of the empty sample holder. Thermogravimetric analysis (TGA) and differential scanning calorimetry were performed by using a Mettler Toledo TGA/DSC1 instrument. Approximately 5 mg of the sample was placed into an alumina crucible and heated from 25–1000 °C at a heating ramp of 10 °C/min under air flow to determine phase breakdown over time and determine the water content of precursor materials for accurate synthesis mass calculations. X-ray fluorescence (XRF) was used to determine the elemental composition of the heavy elements using a Rigaku Primus IV wavelength dispersive spectrometer with a 4 kW Rh X-ray tube. A plastic holder with a flat packed known mass of sample was covered with a 6 μm polypropylene film to confine the thickness of the sample before carrying out a 15 min scan for determination of quantitative elemental makeup. SEM images were collected on a Zeiss Supra 55-VP FEGSEM instrument alongside energy-dispersive X-ray spectroscopy (EDX) measurements using an Oxford Instruments EDX detector.

Magnetometry measurements were performed on a Quantum Designs MPMS-5S SQUID magnetometer. Approximately 30 mg of the sample was placed in a small gel capsule and held in the middle of a plastic straw. Samples were cooled to 5 K in zero field before a field of 1000 Oe was applied. Magnetization vs temperature was measured over the range of 5–300 K, with a ramp rate of 2 °C min^–1^.

X-ray absorption near-edge structure (XANES) spectra were collected in the transmission mode at the Ru K-edge on B18 at the Diamond Light Source, UK.^20^ Samples were combined with cellulose powder and pressed into 13 mm diameter pellets of thickness ∼1 mm to provide absorption in the desired range, and the resulting data were normalized using ATHENA.^21^

For the 3D-ED experiment, the samples were each ground lightly using an agate mortar and pestle and dispersed dry onto copper-supported amorphous carbon TEM grids. These were loaded at room temperature via a JEOL high-tilt specimen holder into a Rigaku XtaLAB Synergy-ED electron diffractometer, operated at 200 kV, and equipped with a Rigaku HyPix-ED hybrid pixel array area detector. Data were collected at 293(5) K as single-rotation scans using CrysAlisPRO (see Table S1.1 for precise versions)^22^ for multiple crystallites appearing as microcrystalline blocks using continuous rotation electron diffraction with a selected area aperture of 2 μm apparent diameter. No indication of beam damage was observed. Data are presented for the most complete and high-quality of these collections, with full experimental details provided in Table S1.1. Data were indexed and integrated with limited scaling and no empirical absorption correction using CrysAlisPRO (version 1.171.44.100a).^22^ The structures were solved using ShelXT^23^ and refined using the Olex2.refine N-beam approximation of dynamical scattering theory as implemented in Olex2 (version 1.5-ac7-014, compiled 2025.02.27 svn.r6f4c0eaf for Rigaku Oxford Diffraction, GUI svn.r7171)^24,25^ using published scattering factors.^26^ O–H protons were refined in the presence of bond length similarity restraints and riding isotropic displacement parameters. The atomic displacement parameters (ADPs) of all metals and equatorial oxygen atoms of trigonal pyramidal Ru centers were refined anisotropically, with constraints on the ADPs of equatorial sites to have the same shape, either globally or within coordination spheres; ADPs of axial oxygens were refined isotropically and constrained to refine together in the same manner. Global rigid bond restraint was employed to improve the physical sense of the anisotropic displacement parameters.

Powder neutron diffraction measurements were performed with two instruments at two different facilities. Time-of-flight data were collected using Polaris^27^ at the ISIS Neutron and Muon Facility, UK, which employs 5 banks of ZnS/Ag/^6^LiF scintillator detectors to access a *d*-spacing range 0.2–13.5 Å. Constant wavelength measurements were made on the D20 diffractometer at the Institut Laue-Langevin, France, using a large microstrip position sensitive detector capable of detecting diffracted neutrons covering a scattering range of 153.6°.^28^ The monochromator used consisted of aligned crystals of highly ordered pyrolytic graphite. With a takeoff angle of 42°, the (002) planes select a wavelength of 2.41 Å, with high incident neutron flux (∼4.2 × 10^7^ s^–1^ cm^–2^). Neutron diffraction was measured from powdered samples held in thin-walled vanadium cans. Analysis of powder diffraction data was carried out using the Rietveld method. For structure refinement of the new materials, heavy-atom (non-H) structure solutions were obtained by 3D-ED and used as the starting models for the program GSAS-II to refine lattice parameters, atomic positions, and thermal parameters.^29^ First, high-resolution synchrotron X-ray data were fitted, and then powder neutron diffraction was used to identify and refine positions of protons in Fourier difference maps to give final structure models (see below). For time-of-flight data from Polaris, simultaneous fits were carried out against data from banks 2–5 over appropriate time-of-flight ranges (*Q*_min_ ∼ 1 Å^–1^, *Q*_max_ ∼ 9 Å^–1^). For constant wavelength data collected at D20, a zero error was refined.

## Results and Discussion

Initially the reaction mixture formed from KRuO_4_ and SrO_2_ (1:2 molar ratio) in water at room temperature was investigated, used previously to crystallize the Ru­(V) oxide SrRu_2_O_6_. The aim was to establish any chemical changes occurring at the earliest stages of crystallization, even before heating. Powder XRD of the resulting solid precipitate, following isolation by filtration after 1 h of stirring the reagents, showed a distinctive and reproducible diffraction pattern (Figure S1) for which a search of the Powder Diffraction File^30^ found a match to the strongest reflections with the material SrRuO_4_·H_2_O (00-035-0949, from a measured pattern reported by Popova et al., assigned as a hexagonal crystal system with *a* = 5.789 Å, *c* = 7.709 Å).^31^ The precipitation was repeated with a 1:1 Ru/Sr as required by the chemical formula, and this yielded a more crystalline sample, with crystallinity improved further by heating the solution to 100 °C and using Sr­(NO_3_)_2_ as a precursor and a 0.5 M NaOH solution as reaction medium. EDXA and XRF analysis confirmed the 1:1 Sr/Ru ratio in the solid product. Under the optical microscope, this optimized polycrystalline sample was seen to consist of submicrometer particles, which was verified when viewed using TEM on the 3D-ED instrument (Figure S2), and hence structure solution was approached using 3D-ED (see Table S1.1). This led to an initial structure model with a monoclinic unit cell in space group *P*2_1_/*n* with Sr, Ru, oxygen, and a single proton located, giving a chemical formula Sr_3_Ru_3_O_14_(OH), with a chemically unlikely oxidation state of Ru larger than +7. TGA of the material showed a mass loss that matched one water molecule per formula unit of the composition SrRuO_4_·H_2_O (Figure S8); hence, powder neutron diffraction (measured on the Polaris diffractometer at ISIS) was used to locate further proton positions via Fourier difference maps and by consideration of bond valence sums of oxygen. The structural model was further confirmed by refinement against high-resolution powder XRD data (Figure S3) and by return to the 3D-ED data. This revealed the chemical formula Sr_3_Ru_3_O_9_(OH)_6_, i.e., SrRuO_3_(OH)_2_, which charge balances with Ru in the +6 oxidation state. Figure 1a shows the final Rietveld fit obtained, which confirms the phase purity of the optimized sample, while crystal structure data and refined parameters are provided in Table S2. Bond distances in the discussion below are taken from the model refined against the neutron powder diffraction data, unless otherwise stated, and atomic labeling is used as per the crystal data presented in Table S2.

**Figure 1:**
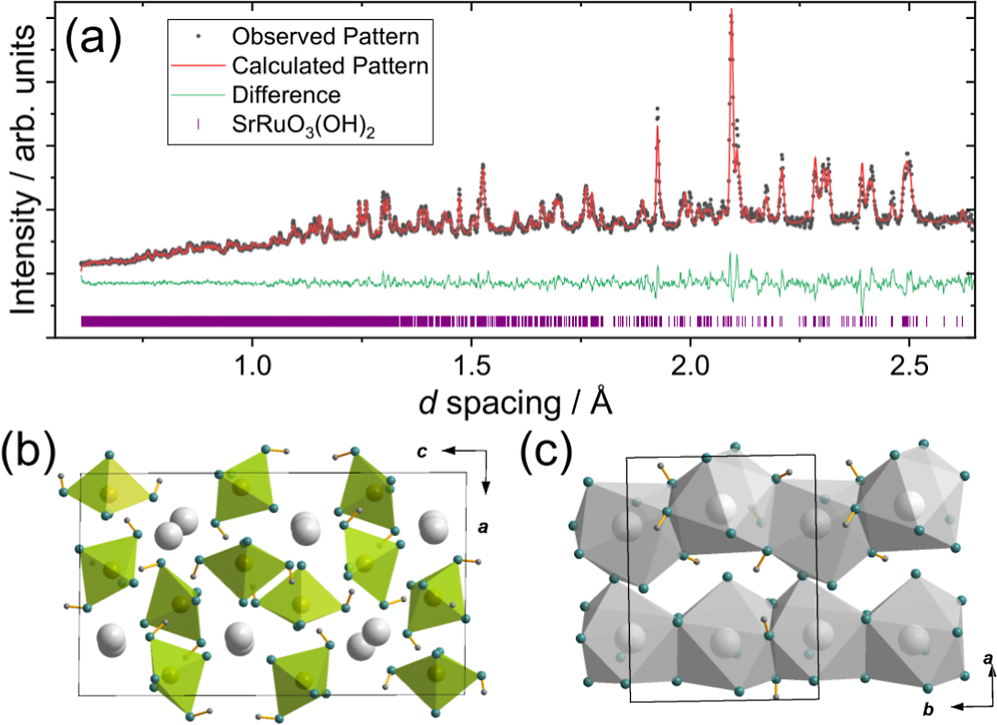
Structural analysis of SrRuO_3_(OH)_2_. (a) Rietveld fit of powder neutron diffraction (time-of-flight on Polaris Bank 5), (b) view of crystal structure showing Ru­(VI)-centered {RuO_3_(OH)_2_} trigonal bipyramids in green, and (c) view of crystal structure showing cross-linked face-shared chains of nine-coordinate Sr polyhedra (gray) with Ru atoms omitted for clarity. Oxygen atoms are colored teal. See Figure S5 for views of the crystal structure with atom labels.

The crystal structure analysis shows that the assumed material SrRuO_4_·H_2_O is actually an oxyhydroxide, SrRuO_3_(OH)_2_. Polyhedral views of its structure are shown in Figure 1b,c that highlight the geometry of the three crystallographically distinct Ru centers, all of which are {RuO_3_(OH)_2_} trigonal bipyramids (Figure 1b), with no shared oxides or hydroxide with other Ru units (the closest Ru–Ru distance is 4.74(1) Å). The OH adopt axial positions of the trigonal bipyramids with longer Ru–O distances (on average 2.02(3) Å) compared to the mean equatorial Ru–O distance to bonded oxides (1.77(5) Å). The hydroxides bridge to strontium such that each OH bridges via oxygen to one Ru and two Sr centers. The equatorial oxide ions around each unique Ru are of two types: one bridges to just one Sr, and the other two bridge to two Sr centers. Three crystallographically distinct Sr centers are present, all of which are nine-coordinated by a mixture of oxide and hydroxide ligands. The coordination environments are irregular with a range of Sr–O distances from 2.49–2.92 Å. Two of the Sr sites are coordinated by six oxides and three hydroxides, while the third is coordinated by six hydroxides and two oxides. The first type shares faces defined by three ligands to create chains of alternating Sr04 and Sr06, with alternating hydroxide and oxide bridging faces, while the second type creates chains of solely Sr05 linked by sharing faces defined by three hydroxide ions. The chains in turn are linked to each other and to the ruthenium centers by shared oxide bridges to form an overall 3D-connected structure, Figure 1c.

There is evidence for hydrogen-bonding interactions between all hydroxides and neighboring oxide anions in SrRuO_3_(OH)_2_, as indicated by oxygen–oxygen distances of less than 3 Å (see Table S1.3). The presence of hydroxide ions was further confirmed by IR spectroscopy, comparing samples prepared in H_2_O and in D_2_O, which showed distinct O–H bands at 3600 cm^–1^ and 3250 cm^–1^ that are shifted to lower wavenumbers by ∼1000 cm^–1^ upon deuteration, and the absence of an H–O–H bending vibration (Figure S9). As noted above, TGA showed evidence for dehydroxylation on heating and in situ XRD with heating revealed that after heating to ∼250 °C, structure collapse commences, with crystallization of SrRuO_3_ perovskite above 350 °C (Figure S10), thus the hydroxide is lost as water.

A barium analogue of this composition, BaRuO_3_(OH)_2_, has previously been reported.^32^ This also contains isolated trigonal bipyramidal {RuO_3_(OH)_2_} units with axially positioned hydroxides and nine-coordinate Ba centers; however, this possesses a different crystal structure, with a hexagonal unit cell, which contains one crystallographically distinct Ba and one Ru center. For this material, the assignment of the axial oxygens as OH groups was based on their longer bond distance (2.04 Å compared to the equatorial distances of 1.76 Å, similar to seen in the Sr material, see above) since the positions of the protons were not detected by the X-ray diffraction experiment used to determine the structure. The environment of the equatorial oxide anions differs from SrRuO_3_(OH)_2_ since all bridge to two alkali-earth cations. The nine-coordinate Ba centers share triangular faces to create chain motifs, but unlike in SrRuO_3_(OH)_2_, the chains are not connected directly by shared oxide or hydroxide between alkali earth cations (see Figure S7). It is also worth noting that Popova et al. also reported a phase assigned as “CaRuO_4_·2H_2_O”,^31^ whose structure to our knowledge has never been resolved, and for which Johnson later suggested that its powder pattern actually matched Ca­(OH)_2_,^33^ such that its existence must be questioned.

Having prepared a Ru­(VI) material under mild conditions, the possibility that Sr-rich conditions might also provide a method of stabilizing Ru­(VI) in oxides was considered, based on the inductive effect expected by using electropositive cations as partners in a ternary oxide.^14^ We thus explored hydrothermal reactions of KRuO_4_ with an excess of Sr^2+^ in synthesis, under otherwise identical conditions used to prepare SrRu_2_O_6_. This reproducibly formed a novel material with a distinct powder XRD pattern with molar ratios Sr/Ru > 2, while at ratios 2 > Sr/Ru > 1, coexistence with the phase Sr_2_Ru_3_O_10_ reported by Marchandier et al. was observed (see Figure S11).^11^ Structure solution of the new material was accomplished with 3D-ED followed by refinement of the initial model, including location of the protons, using powder neutron diffraction, Figure 2a to give a final crystal structure (Table S1.3). This revealed the composition Sr_3_Ru_2_O_8_(OH)_2_, which, as anticipated, contains Ru­(VI) and an excess of strontium compared to ruthenium. The final model also provides a good fit to high-resolution synchrotron powder X-ray diffraction data (Figure S4).

**Figure 2:**
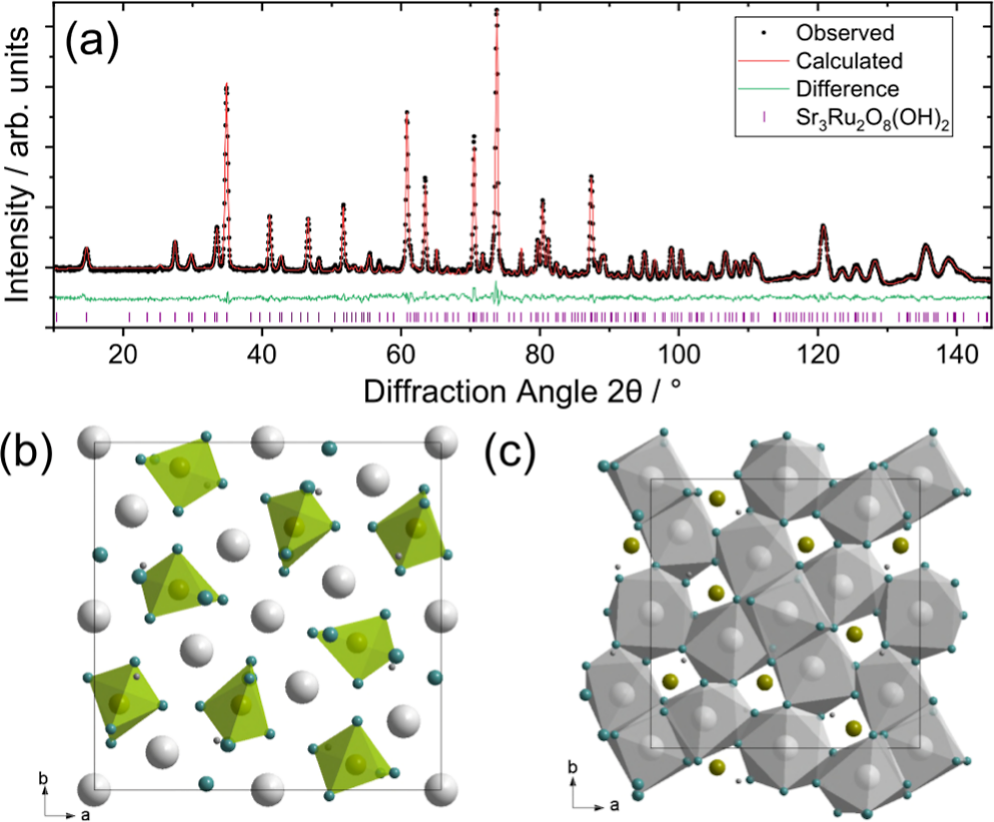
Structural analysis of Sr_3_Ru_2_O_8_(OH)_2_. (a) Rietveld fit of powder neutron diffraction (fixed wavelength, λ = 2.41 Å on D20), (b) view of crystal structure showing Ru­(VI)-centered {RuO_4_(OH)} trigonal bipyramids in green, and (c) view of crystal structure showing cross-linked edge- and corner-shared eight-coordinate Sr polyhedra (gray). See Figure S6 for views of the crystal structure with atom labels.

Sr_3_Ru_2_O_8_(OH)_2_ (tetragonal *P*4̅, *a* = 13.2206(5) Å *c* = 5.4852(2) Å) contains two crystallographically distinct Ru sites. These are both {RuO_4_(OH)} trigonal bipyramids, with no oxides or hydroxide shared with other Ru units, Figure 2b. As with the first material, the mean axial Ru–O distance (2.04(6) Å) is longer than the mean equatorial distance (1.77(7) Å), while the closest Ru–Ru distance is 4.95(2) Å. The OH again adopts axial positions and bridges to strontium such that each OH bridges 1 Ru and 2 Sr centers, while the oxide anions connect to either two or three Sr centers. There are five unique Sr sites (note these have various crystallographic multiplicity such that the Sr/Ru ratio is 3:2), all of which are coordinated by eight O/OH, having irregular coordination with Sr–O distances ranging from 2.35 to 2.9 Å. These polyhedra share a combination of edges and corners to create a complex three-dimensional structure, Figure 2c.

TGA and X-ray thermodiffractometry together confirmed the composition of the material and its collapse at ∼400 °C to give Sr_4_Ru_2_O_9_ and RuO_2_ as the crystalline products, which remain to 900 °C (Figure S12). IR spectroscopy confirms the presence of O–H bands due to hydroxide (Figure S13). Unlike SrRuO_3_(OH)_2_, the crystal structure of Sr_3_Ru_2_O_8_(OH)_2_ does not provide any evidence for hydrogen bonding of the hydroxide ions to any oxygen atoms in its vicinity. The IR spectra of the two materials, while both show sharp and distinctive O–H stretching bands in the region 3600–3000 cm^–1^, are also markedly different, consistent with the different environments of the hydroxide anion in the two materials. In particular, SrRuO_3_(OH)_2_ shows a red-shifted O–H stretch in comparison to Sr_3_Ru_2_O_8_(OH)_2_, which is indicative of hydrogen-bonded hydroxide in the former.^34^

Further characterization of the two new strontium ruthenium oxyhydroxides was performed to confirm the presence of Ru­(VI). Figure 3a shows XANES spectra recorded at the Ru K-edge in comparison to spectra from oxides containing Ru­(IV), Ru­(V) and Ru­(VII). The degree of edge shift (Figure 3b) correlates with the oxidation state of ruthenium, with a shift of ∼1.1 eV per oxidation state unit (with the edge position defined as the energy at which normalized absorption is 0.5), and this is consistent with previous literature on Ru K-edge XANES spectra.^10^ In addition, the Ru­(VI) spectra both have significant pre-edge features consistent with the noncentrosymmetric coordination environment of Ru in trigonal bipyramidal geometry, which is otherwise very weak in RuO_2_ and SrRu_2_O_6_ that contain close to ideal octahedral coordination for Ru. Magnetization measurements and analysis by a Curie–Weiss fit to the linear region of the temperature-dependent inverse susceptibility plot show that SrRuO_3_(OH)_2_ (Figure 3c) has a Curie constant of 0.891(1) emu K mol^–1^ and a Weiss temperature of −71.6(8) K giving a μ_eff_ Ru^–1^ of 2.67(9) μ_B_, and Sr_3_Ru_2_O_8_(OH)_2_ (Figure 3d) has a Curie constant of 0.920(1) emu K mol^–1^ Ru and Weiss temperature of −85.4(2) K giving a μ_eff_ Ru^–1^ of 2.71(7) μ_B_. Both values are close to the expected value for two unpaired electrons of 2.83 μB, and by inspection of the crystal field splitting of a trigonal bipyramid, as described by Companion and Komarynsky,^35^ this is consistent with the 4d^2^ configuration expected for Ru­(VI). Similar magnetic behavior was reported for K_2_RuO_3_(OH)_2_, where a μ_eff_ Ru^–1^ of 2.721 (3) μ_B_ was found.^36^ As noted above, in both of the new materials studied here, the Ru­(VI) centers are separated by >4.7 Å with no shared oxygen atoms, so there is no possibility of magnetic exchange and cooperative magnetism.

**Figure 3:**
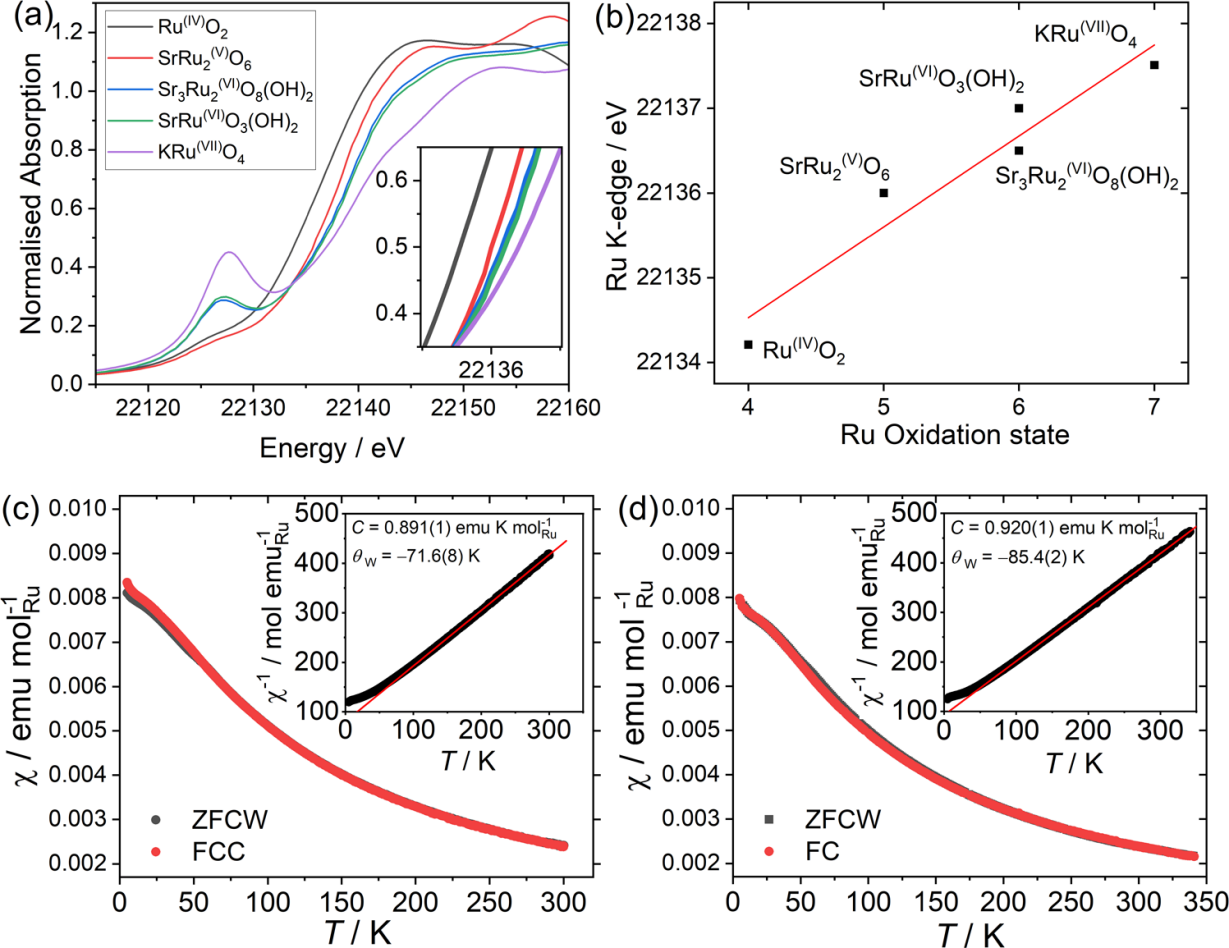
Characterization of ruthenate (VI) materials. (a) Ru K-edge XANES spectra for the ruthenium (VI) oxyhydroxides and Ru­(IV), Ru­(V), and Ru­(VII) reference materials. (b) Ru K-edge position as a function of oxidation state with a linear fit to edge positions of reference materials. Magnetic susceptibility vs temperature for (c) SrRuO_3_(OH)_2_ and (d) Sr_3_Ru_2_O_8_(OH)_2_. ZFCW = zero field cooled warming, and FC = field cooled. The insets in (c) and (d) show Curie–Weiss fits to the paramagnetic region (>125 K) of inverse susceptibility, with derived magnetic parameters indicated.

It is informative to compare the local structure of the two new materials with other Ru­(VI) oxides and oxyhydroxides reported in the literature, and Table 1 contains relevant information along with the synthesis conditions used in the preparation of the materials. This reveals that trigonal bipyramidal coordination Ru­(VI) is not uncommon in ternary oxides and oxyhydroxides, and in the case when hydroxide is present, it is always coordinated to the Ru center, always in an axial position. In terms of the previous synthesis methods for preparing Ru­(VI) oxides and oxyhydroxides, the majority reported make use of elevated temperature using sealed reaction vessels with either oxygen atmosphere or the use of peroxides as reagents. The reason for this may have been to access crystals suitable for structure determination since the aqueous precipitations described are more likely to yield polycrystalline powders, as in the current work. Nevertheless, our new work highlights that mild synthesis routes can also be used to stabilize Ru­(VI) compounds in the absence of strongly oxidizing conditions.

**Table 1:** Summary of Synthesis Condition and Local Atomic Environment of Ru in Reported Ruthenium (VI) Oxides and Oxyhydroxides

material	synthesis method	Ru coordination environment(s)
BaRuO_3_(OH)_2_	RuO_4_ or KRuO_4_ in alkali solution in the presence of BaSO_4_ or other insoluble Ba salt^32^	trigonal bipyramidal {RuO_3_(OH)_2_}
K_2_RuO_3_(OH)_2_	Ru and KNO_3_ in molten KOH in a nickel crucible,^36^ or evaporating an aqueous extract of a KOH melt with RuO_2_ ^37^	trigonal bipyramidal {RuO_3_(OH)_2_}
Na_2_RuO_4_	Na_2_O_2_ and RuO_2_ heated under oxygen pressure up to 900 K^38,39^	trigonal bipyramidal {RuO_5_} (corner shared)
BaHgRuO_5_	Ru, HgO, BaO at 600 °C 5000 bar oxygen pressure oxygen stream at 898 K^40^	trigonal bipyramidal {RuO_5_}
SrRuO_3_(OH)_2_	KRuO_4_, Sr(NO_3_)_2_ in water room temperature (this work)	trigonal bipyramidal {RuO_3_(OH)_2_}
Sr_3_Ru_2_O_8_(OH)_2_	KRuO_4_, 2Sr(NO_3_)_2_, 0.5 M NaOH, 200 °C hydrothermal (this work)	trigonal bipyramidal {RuO_4_(OH)}
Cs_2_RuO_4_	Cs_2_O_2_, RuO_2_ in a sealed Pd tube at 800 °C^15^	tetrahedral {RuO_4_}
K_2_RuO_4_	K_2_O_2_, RuO_2_ in a Pd crucible at 780 °C^41^	tetrahedral {RuO_4_}
Rb_2_RuO_4_	RbO_1.6_ + RuO_2_ in a Pd crucible at 650 °C^41^	tetrahedral {RuO_4_}
K_3_Na(RuO_4_)_2_	RuO_2_, KO_2_, Na_2_O_2_ at 873 K under 175 MPa oxygen pressure^42^	tetrahedral {RuO_4_}
Rb_3_Na(RuO_4_)_2_	RuO_2_, RbO_2_, Na_2_O_2_ 873 K under 175 MPa oxygen pressure^42^	tetrahedral {RuO_4_}
CsK_5_Ru_2_O_9_	KO_1.1_ + CsO_1.2_ + RuO_2_ Ag-tube 750 °C^16^	tetrahedral and trigonal bipyramidal {RuO_4_} and {RuO_5_}
CuRuO_2_(OH)_4_	precipitation from KRuO_4_ with Cu^2+^ (aq) solution^18^	octahedral {Ru(OH)_4_O_2_} (shared site with Jahn–Teller distorted Cu^2+^)
Na_5_K[RuO_2_(HIO_6_)_2_]·8H_2_O	RuO_2_ + KOH/KOCl (aq) + H_5_IO_6_/NaOH (aq) room temperature^43^	octahedral {RuO_6_} with terminal *trans* oxo groups

Figure 4 compares the trigonal-bipyramidal environments of Ru in the two new materials. For the materials reported here, the bond valence sum (BVS) method was used as a final verification of the Ru-oxidation state. Initially, we used reported BVS parameters to derive Ru valence, but since these may not have accounted fully for the range of Ru­(VI) materials reported in the literature, we used the materials in Table 1, to derive bond valence parameters that accounted for Ru­(VI) in the new materials. The values calculated for the two new materials are reported in Table 2, while the Supporting Information contains further details for all the materials considered in this analysis (Table S5). This confirms that the crystal structures of SrRuO_3_(OH)_2_ and Sr_3_Ru_2_O_8_(OH)_2_ are consistent with the presence of Ru­(VI).

**Figure 4:**
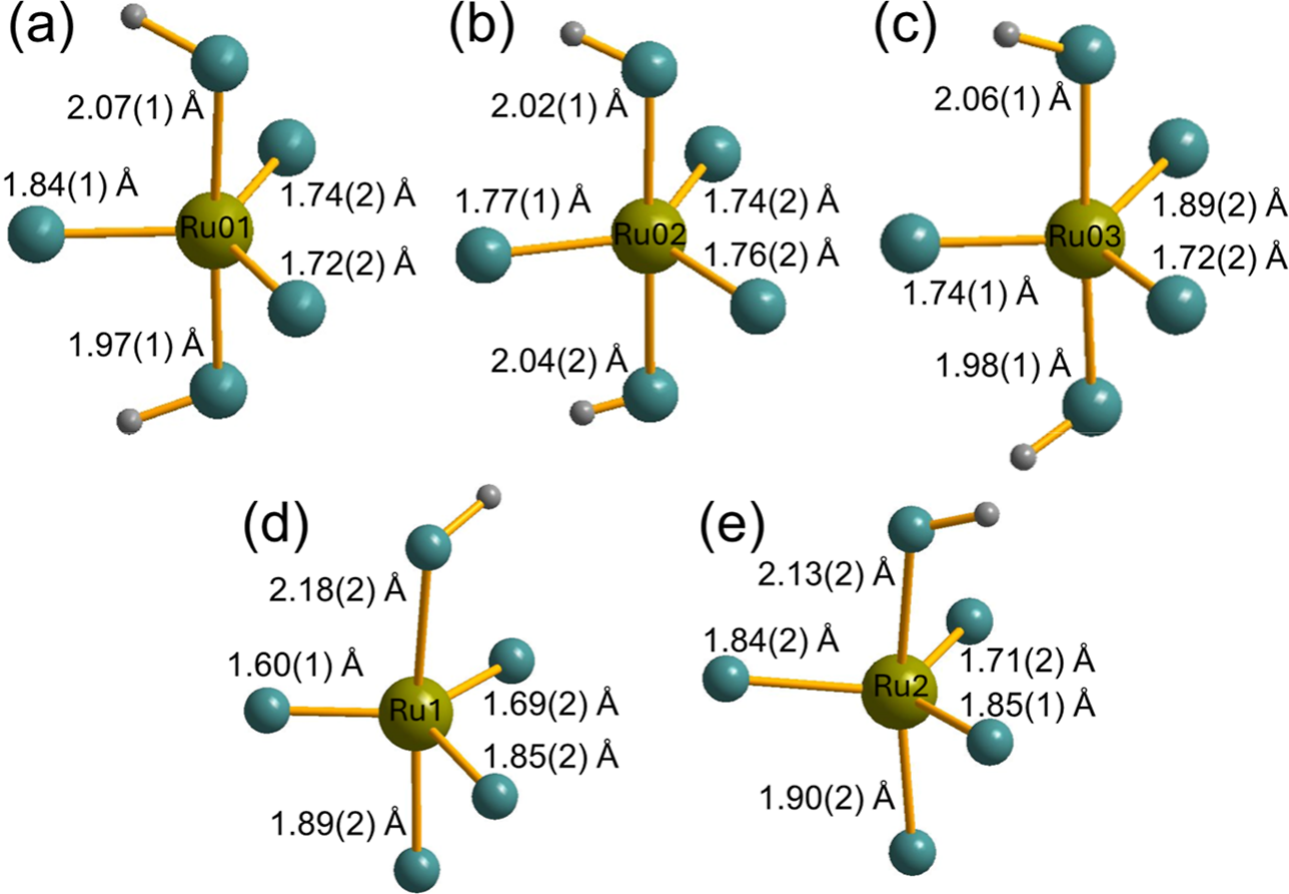
Local environment of Ru in SrRuO_3_(OH)_2_, (a–c), and in Sr_3_Ru_2_O_8_(OH)_2_, (d,e) with Ru–O bond distances indicated.

**Table 2:** Results of BVS Analysis for Ru in SrRuO_3_(OH)_2_ and Sr_3_Ru_2_O_8_(OH)_2_ ^a^

material	site	BVS
SrRuO_3_(OH)_2_	Ru01	5.97
	Ru02	6.00
	Ru03	5.78
Sr_3_Ru_2_O_8_(OH)_2_	Ru1	6.87
	Ru2	5.73

a See Supporting Information for BVS parameters.

## Conclusions

The use of mild aqueous synthesis conditions from the high oxidation state precursor potassium ruthenate (VII) allows the isolation of two strontium ruthenate (VI) materials. Structure solution reveals trigonal bipyramidal coordination for Ru in both materials with hydroxide on axial positions. Comparison with the literature shows that other ruthenate (VI) materials exhibit this local coordination, although not exclusively. The two materials add to a set of ruthenates that contain the element in the +6 oxidation state, for which previous examples have typically been prepared in highly oxidizing environments. Our results point to the possible discovery of further new materials using mild aqueous chemistry as a more convenient synthesis approach compared to other methods, where typically peroxide fluxes or oxygen pressure have been employed for the isolation of high oxidation states of precious metals in oxides and oxyhydroxides.

## Supplementary Material



## Data Availability

Neutron scattering data from ISIS and the ILL are available at the following urls, respectively: https://doi.org/10.5286/ISIS.E.RB2220557-1, https://doi.ill.fr/10.5291/ILL-DATA.5-24-665. Experimental and refinement information are contained within the deposited CIFs which can be obtained free of charge from The Cambridge Crystallographic Data Centre via www.ccdc.cam.ac.uk/structures with the following CSD numbers: 2469818 SrRuO_3_(OH)_2_ refined structure from 3DED, 2469819 Sr_3_Ru_2_O_8_(OH)_2_ refined structure from 3DED, 2469909 SrRuO_3_(OH)_2_ refined structure from powder neutron diffraction, and 2469910 Sr_3_Ru_2_O_8_(OH)_2_ refined structure from powder neutron diffraction.

## Notes

Further characterization data including details of crystallography, powder XRD, TGA, IR spectra, and X-ray thermodiffractometry, and further description of crystal structures and BVS analysis (PDF)
