# Supplementary material for: Aqueous Synthesis of Strontium Ruthenate(VI) Oxyhydroxides and Their Crystal Structure Solution from Microcrystals
Source: Inorg Chem. 2025 Sep 1;64(36):18471–8. doi: 10.1021/acs.inorgchem.5c03066 (PMC12442060; doi:10.1021/acs.inorgchem.5c03066)
Supplement: Supplementary file 1 [file ic5c03066_si_001.pdf]

SUPPORTING INFORMATION

Aqueous Synthesis of Strontium Ruthenate (VI) Oxyhydroxides and Their Crystal Structure Solution from Microcrystals

Mark Crossman,[1] Craig I. Hiley,[1] Helen Y. Playford,[2] Ronald I. Smith,[2] Thomas C. Hansen,[3] Jeremiah P. Tidey,[4] and Richard I. Walton[1,*]

1. Department of Chemistry, University of Warwick, Gibbet Hill Road, Coventry CV4 7AL, UK *Author to whom correspondence should be addressed: r.i.walton@warwick.ac.uk

2. ISIS Facility, Rutherford Appleton Laboratory, Didcot, OX11 0QX, UK

3. Institut Laue-Langevin, 71 Avenue des Martyrs CS 20156, 38042 Grenoble Cedex 9, France.

4. Department of Physics, University of Warwick, Gibbet Hill Road, Coventry CV4 7AL, UK

S1: Crystal structure analysis

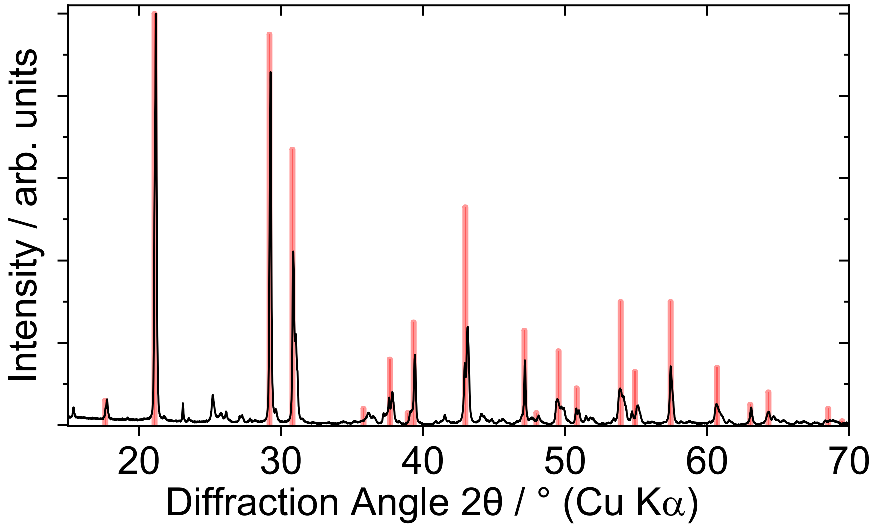

Figure S1: Powder XRD of precipitated '$SrRuO_4.H_2O$' compared with Powder Diffraction File 00-035-0949 (from Popova *et al.* [1])

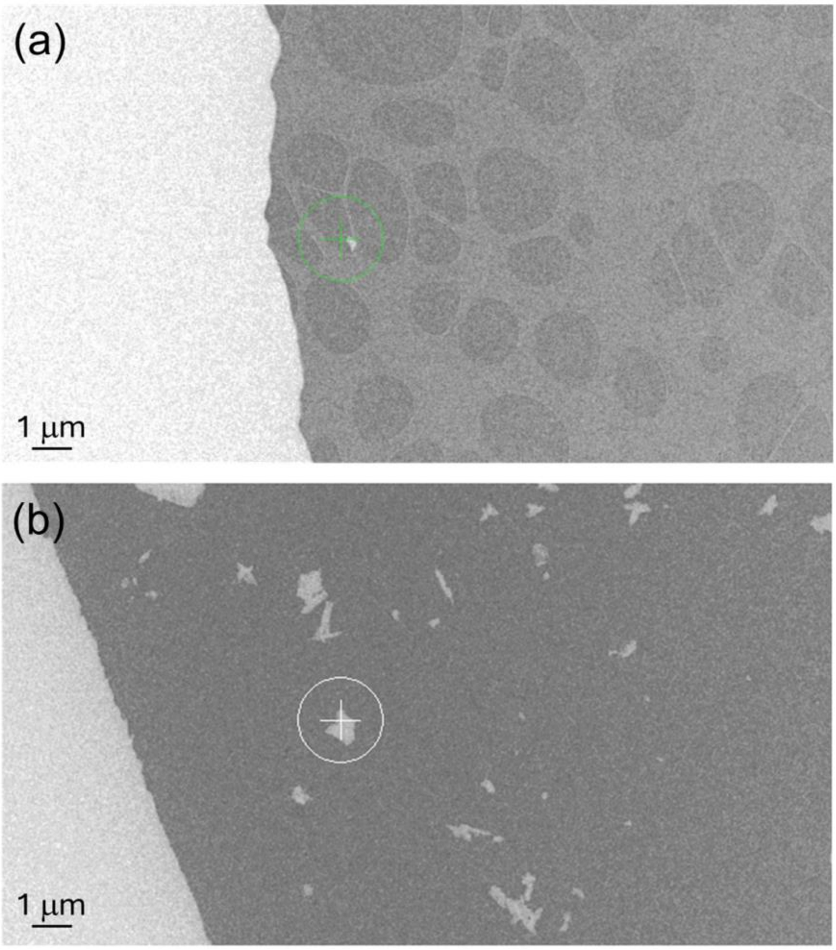

Figure S2: Images of TEM grid for 3D-ED for (a) $SrRuO_3(OH)_2$ and (b) $Sr_3Ru_2O_8(OH)_2$ recorded on the 3D-ED using defocused diffraction mode.

Table S1: 3D-ED Experimental data details

	$Sr_3Ru_2O_8(OH)_2$	$Sr_3Ru_3O_9(OH)_6$
M_r	627.01	811.86
Crystal system, space group	Tetragonal, $P\bar{4}$	Monoclinic, $P2_1/n$
Temperature (K)	293(5)	293(5)
a, b, c (Å)	13.1177(14), 13.1177(14), 5.4601(9)	9.924(4), 7.653(2), 17.252(11)
α, β, γ (°)	90, 90, 90	90, 90.54(5), 90
V (Å^3)	939.5(2)	1310.2(11)
Z	4	4
Radiation type	Electron, λ = 0.02510 Å	Electron, λ = 0.02510 Å
**Data collection**		
Frame width (°)	0.25	0.5
Scan range (°)	85.25	105
h_{min}, h_{max}	−16, 16	−13, 13
k_{min}, k_{max}	−16, 16	−11, 11
l_{min}, l_{max}	−6, 6	−23, 22
Diffractometer	XtaLAB Synergy-ED, HyPix-ED, electron source at 200keV	
Data collection	*CrysAlis PRO* system 1.171.43.75a (released 14-06-2023)	*CrysAlis PRO* system 1.171.43.94a (released 20-10-2023)
Integration and scaling	*CrysAlis PRO* 1.171.44.100a (Rigaku Oxford Diffraction, 2025) SCALE3 ABSPACK scaling; no empirical absorption correction.	

measured, independent, observed [$I \geq 2u(I)$] reflections	3800, 3800, 2637	9155, 9155, 2682
R_{int}	0.193	0.241
$(\sin \theta/\lambda)_{max}$ (Å^{-1})	0.640	0.737
**Refinement**		
R_1, wR_2 ([$F^2 > 2\sigma(F^2)$])	0.1405, 0.3278	0.1538, 0.3845
R_1, wR_2 (all)	0.1577, 0.3436	0.2766, 0.4871
GoF(S) (inc., excl. restraints)	1.0694, 1.0703	0.8824, 0.8831
data, parameters, restraints	2040, 90, 6	2590, 140, 15
$\Delta\varphi_{max}$, $\Delta\varphi_{min}$ (as reported by Olex2.refine, AC07-014)	8.76, −3.18	8.67, −4.83

Table S2: Tables of final refined crystal parameters from powder neutron diffraction for $SrRuO_3(OH)_2$

Space group			$P\ 1\ 2_1/n\ 1$		
a **/ Å**			9.9903(3)		
b **/ Å**			7.7023(2)		
c **/ Å**			17.3677(6)		
**β / °**			89.353(2)		
V **/ Å^3**			1336.3(1)		
**Atom**	**Wyckoff**	***x*/*a***	***y*/*b***	***z*/*c***	**U_{iso} / Å^2**
Ru01	4*e*	0.5906(8)	0.2686(15)	0.7375(5)	0.0055(8)
Ru02	4*e*	0.4055(10)	0.7435(14)	0.5738(5)	0.0055(8)
Ru03	4*e*	0.4352(8)	0.7279(15)	0.9165(5)	0.0055(8)
Sr04	4*e*	0.7349(9)	0.0090(11)	0.5857(5)	0.0043(6)
Sr05	4*e*	0.7605(9)	0.5033(11)	0.5825(5)	0.0043(6)
Sr06	4*e*	0.2168(10)	0.4972(15)	0.7304(5)	0.0043(6)
O007	4*e*	0.5360(12)	0.0796(17)	0.6954(6)	0.0058(5)
O008	4*e*	0.3083(13)	0.9339(17)	0.5659(7)	0.0058(5)
O009	4*e*	0.2751(11)	0.712(2)	0.8504(7)	0.0058(5)
O00A	4*e*	0.0962(12)	0.7938(19)	0.7108(6)	0.0058(5)
O00C	4*e*	0.3908(11)	0.7396(19)	0.6897(6)	0.0058(5)
O00D	4*e*	0.4935(11)	0.9241(18)	0.8591(7)	0.0058(5)
O00E	4*e*	0.7636(10)	0.242(2)	0.6836(6)	0.0058(5)
O00F	4*e*	0.5743(11)	0.4754(18)	0.6978(6)	0.0058(5)
O00G	4*e*	0.6763(11)	0.258(2)	0.8308(5)	0.0058(5)
O00H	4*e*	0.3172(12)	0.5486(18)	0.5741(7)	0.0058(5)
O00J	4*e*	0.3408(11)	0.738(2)	1.0015(6)	0.0058(5)
O00K	4*e*	0.4153(11)	0.723(2)	0.4565(7)	0.0058(5)
O00L	4*e*	0.4941(11)	0.5264(18)	0.8898(6)	0.0058(5)
O00M	4*e*	0.6061(10)	0.7666(19)	0.9797(6)	0.0058(5)
O00N	4*e*	0.5824(11)	0.748(2)	0.5747(6)	0.0058(5)
H00D	4*e*	0.070(3)	0.864(3)	0.6598(16)	0.0422(26)
H00F	4*e*	0.489(2)	0.725(4)	0.4495(13)	0.0422(26)
H00H	4*e*	0.830(2)	0.217(3)	0.7229(13)	0.0422(26)
H00I	4*e*	0.465(2)	0.681(3)	0.7054(13)	0.0422(26)
H00G	4*e*	0.212(2)	0.658(3)	0.8781(14)	0.0422(26)
H00E	4*e*	0.597(2)	0.744(5)	1.0331(13)	0.0422(26)

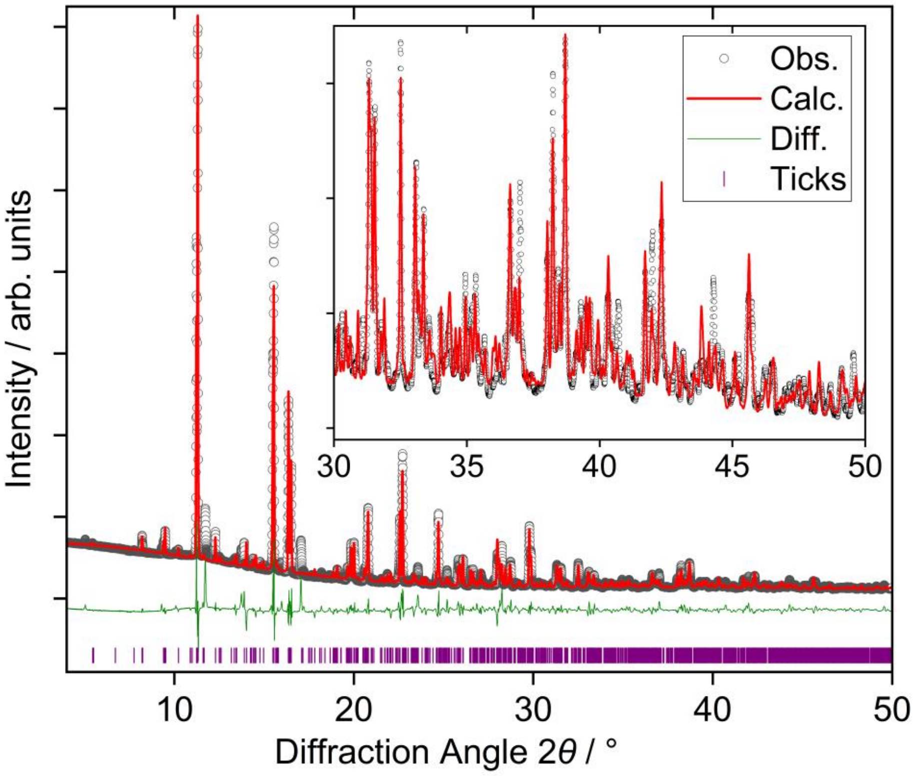

Figure S3: **Rietveld fit of final crystal structure model against high-resolution powder XRD (λ = 0.825960(1) Å) to confirm the structural model for $SrRuO_3(OH)_2$**

Table S3: Likely hydrogen bonded interactions in $SrRuO_3(OH)_2$

**OH**	**O**	**Distance /Å**
O00C	O00F	2.74(2)
O00K	O00H	2.63(2)
O00A	O00L	2.67(2)
O00E	O00F	2.62(2)
O00M	O008	2.96(2)
O009	O008	2.71(2)

Table S4: Final refined crystal parameters from powder neutron diffraction for $Sr_3Ru_2O_8(OH)_2$

**Space group**			$P\bar{4}$		
***a* / Å**			13.2206(5)		
***c* / Å**			5.4852(2)		
***V* / Å^3**			958.72(9)		
**Atom**	**Wyckoff**	***x*/*a***	***y*/*b***	***z*/*c***	**U_{iso} / Å^2**
Ru1	4*h*	0.0777(9)	0.2531(8)	0.4396(19)	0.0202(15)
Ru2	4*h*	0.2489(8)	0.5731(8)	0.0724(19)	0.0202(15)
Sr1	1*b*	0	0	0.5	0.0189(15)
Sr5	1*c*	0.5	0.5	0	0.0189(15)
Sr2	4*h*	-0.1061(9)	0.2012(9)	0.0047(22)	0.0189(15)
Sr4	4*h*	0.2950(8)	0.3961(8)	0.4975(25)	0.0189(15)
Sr3	2*g*	0	0.5	0.5473(31)	0.0189(15)
O11	4*h*	0.0571(9)	0.1723(10)	0.6727(19)	0.0150(8)
O8	4*h*	0.3092(9)	0.5446(11)	-0.2233(16)	0.0150(8)
O2	4*h*	0.0524(10)	0.1449(10)	0.2231(21)	0.0150(8)
O4	4*h*	0.1295(10)	0.3772(9)	0.6686(23)	0.0150(8)
O7	4*h*	0.3655(9)	0.5386(11)	0.2577(18)	0.0150(8)
O6	4*h*	0.1574(9)	0.4788(10)	0.1852(20)	0.0150(8)
O3	4*h*	0.2078(8)	0.2492(13)	0.3141(20)	0.0150(8)
O5	4*h*	-0.0061(11)	0.3209(9)	0.3067(21)	0.0150(8)
O10	4*h*	0.2499(11)	0.6913(8)	0.1975(19)	0.0150(8)
O9	4*h*	-0.1391(10)	0.3925(9)	-0.1986(25)	0.0150(8)
H1	4*h*	0.1610(18)	0.6398(15)	0.666(4)	0.029(4)
H2	4*h*	0.1335(14)	0.3510(16)	0.828(4)	0.029(4)

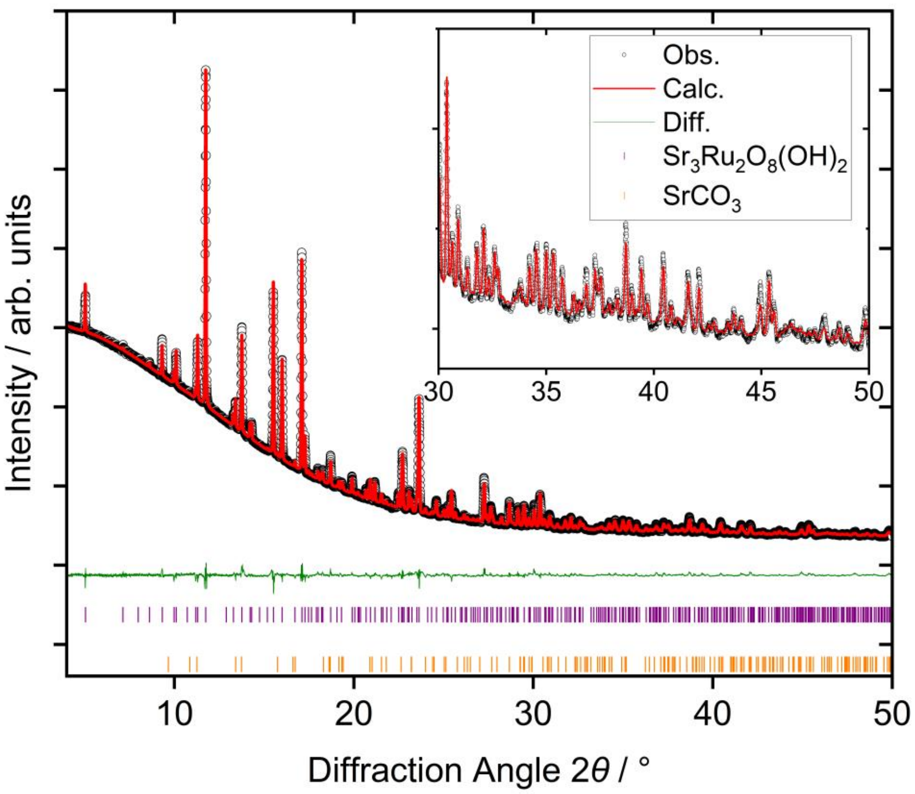

Figure S4: **Rietveld fit of final crystal structure model against high-resolution powder XRD (λ = 0.825960(1) Å) to confirm the structural model for $Sr_3Ru_2O_8(OH)_2$**

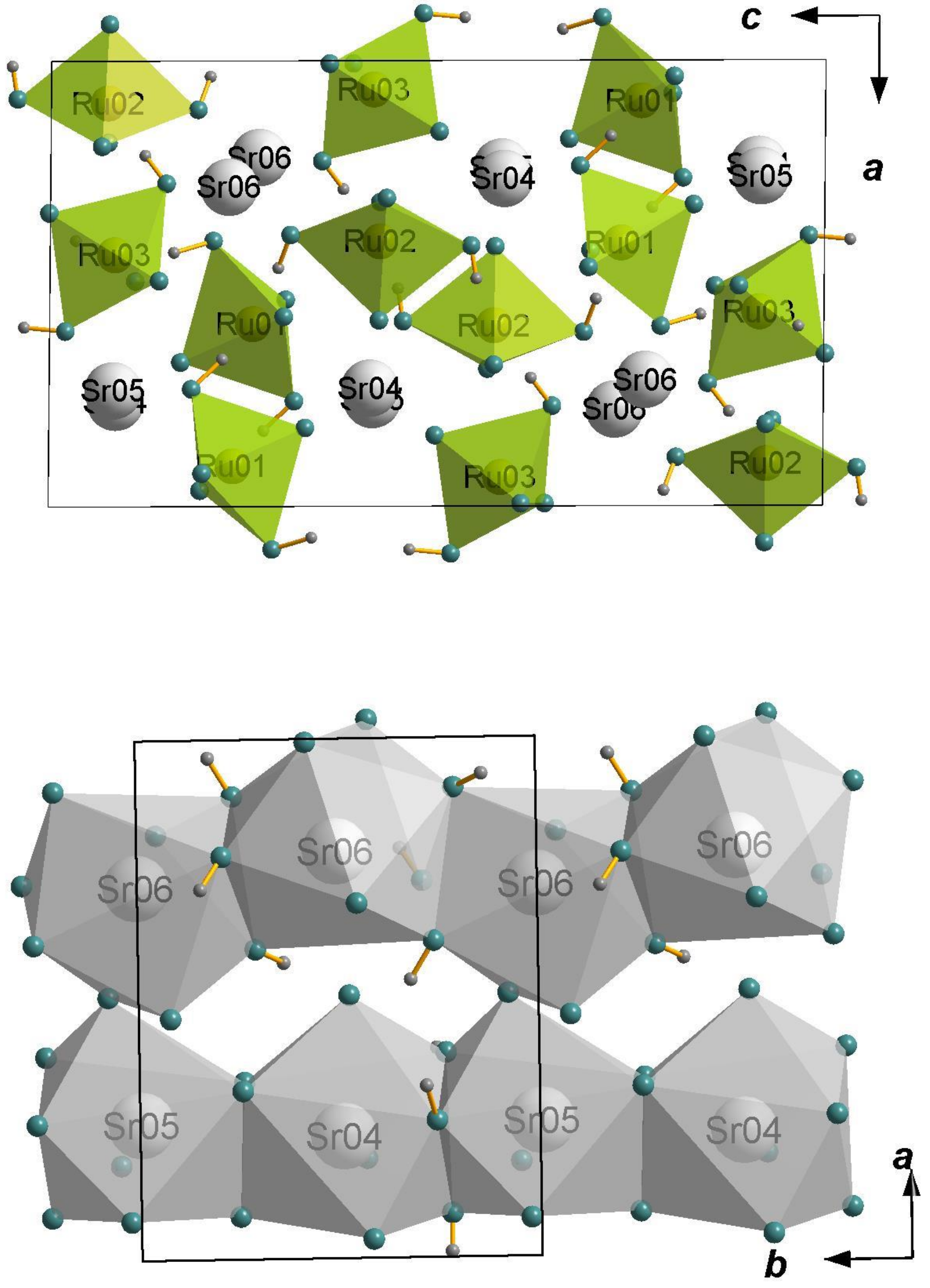

Figure S5: Views of the crystal structure of $SrRuO_3(OH)_2$ with atom labels. Top: view of crystal structure showing Ru(VI)-centred $\{RuO_3(OH)_2\}$ trigonal bipyramids in green, Bottom: view of crystal structure showing cross-linked face-shared chains of eight-coordinate Sr polyhedra (grey) with Ru atoms omitted for clarity. Oxygen atoms are coloured teal.

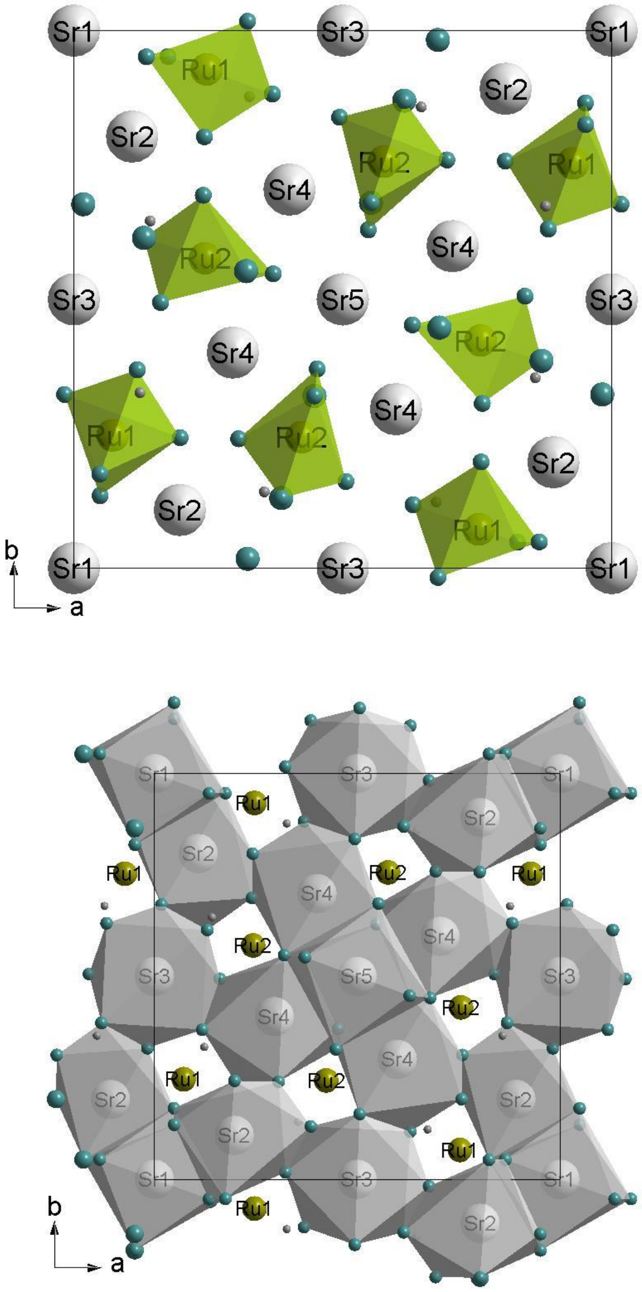

Figure S6: Views of the crystal structure of $Sr_3Ru_2O_8(OH)_2$. Top: view of crystal structure showing Ru(VI)-centred {$RuO_4(OH)$} trigonal bipyramids in green. Botton: view of crystal structure showing cross-linked edge- and corner-shared eight-coordinate Sr polyhedra (grey).

Table S5: Bond valence sum (BVS) analysis of Ru(VI) materials using the literature values (IUCr: http://www.iucr.org/resources/data/datasets/bond-valence-parameters), and recalculated values by analysis of structures published in the literature.

Material	Site	BVS R_{ij} = 1.87 Å<br>b = 0.35 (IUCr)	BVS R_{ij} = 1.912 Å<br>b = 0.37<br>(this work)
$BaRuO_3(OH)_2$ [2]	Ru1 trigonal bipyramidal	6.04	6.03
$K_2RuO_3(OH)_2$ [3]	Ru1 trigonal bipyramidal	6.02	6.02
$K_2RuO_3(OH)_2$ [4]	Ru1 trigonal bipyramidal	6.04	6.03
Na_2RuO_4 [5]	Ru1 trigonal bipyramidal<br>Ru2 trigonal bipyramidal	5.93<br>5.85	5.93<br>5.85
Na_2RuO_4[6]	Ru1 trigonal bipyramidal<br>Ru2 trigonal bipyramidal	6.02<br>6.03	6.02<br>6.02
$BaHgRuO_5$ [7][a]	Ru1 trigonal bipyramidal	7.22	7.17
Cs_2RuO_4 [8]	Ru1 tetrahedral	6.06	5.99
K_2RuO_4 [9][b]	Ru1 tetrahedral	6.76	6.65
Rb_2RuO_4 [9]	Ru1 tetrahedral	6.09	6.02
$K_3Na(RuO_4)_2$ [10]	Ru1 tetrahedral	6.07	6.01
$Rb_3Na(RuO_4)_2$ [10]	Ru1 tetrahedral	6.04	6.00
$CsK_5Ru_2O_9$ [11]	Ru1 tetrahedral<br>Ru1 trigonal bipyramidal	6.24<br>5.95	6.16<br>5.93
$CuRuO_2(OH)_4$ [12][c]	Ru1 octahedral	6.73	6.72
$SrRuO_3(OH)_2$<br>(this work)	Ru1 trigonal bipyramidal<br>Ru2 trigonal bipyramidal<br>Ru3 trigonal bipyramidal	5.37<br>5.40<br>5.18	5.97<br>6.00<br>5.78
$Sr_3Ru_2O_8(OH)_2$<br>(this work)	Ru1 trigonal bipyramidal<br>Ru2 trigonal bipyramidal	4.92<br>5.95	6.87<br>5.73

a: The crystal structure contains abnormally short Ru-O distances so this was excluded from the redetermination of the BVS parameters.

b: The crystal structure contains large displacement parameters for oxygens, as noted by the authors, so this was excluded from the redetermination of the BVS parameters.

c: In this material Ru^{6+} is on shared site with Jahn-Teller distorted Cu^{2+} so this was excluded from the redetermination of the BVS parameters.

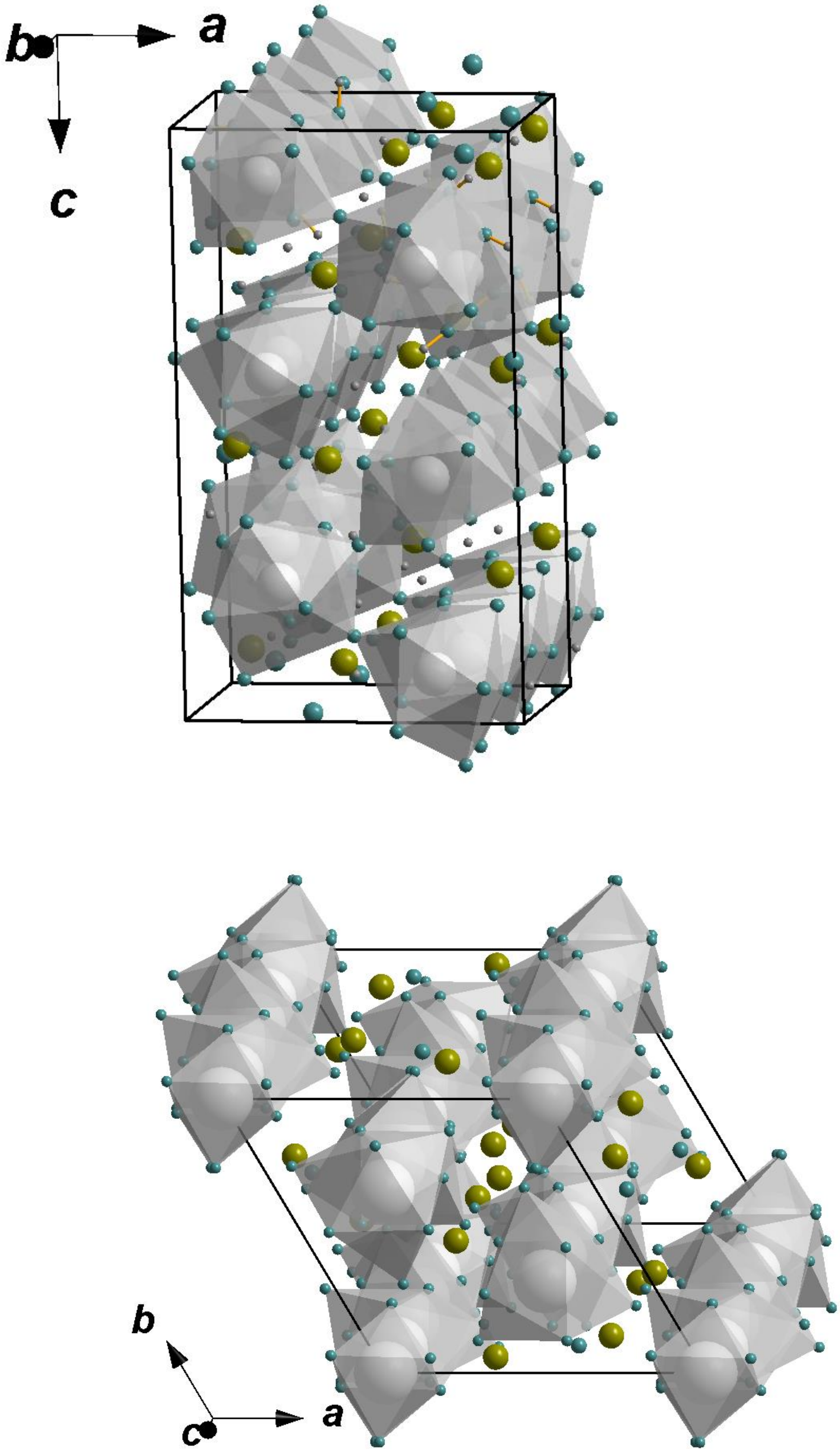

Figure S7: Comparison of the crystal structures of: (top) $SrRuO_3(OH_2)$ (monoclinic, $P2_1/n$) and (bottom) $BaRuO_3(OH_2)$ (trigonal, $R\overline{3}c$) [2]. The structures are viewed so to highlight the cross-linked chains of face-shared Sr-centred polyhedra in $SrRuO_3(OH_2)$ and the non-connected chains in $BaRuO_3(OH_2)$. Ru atoms are coloured olive and O teal.

S2: Characterisation data

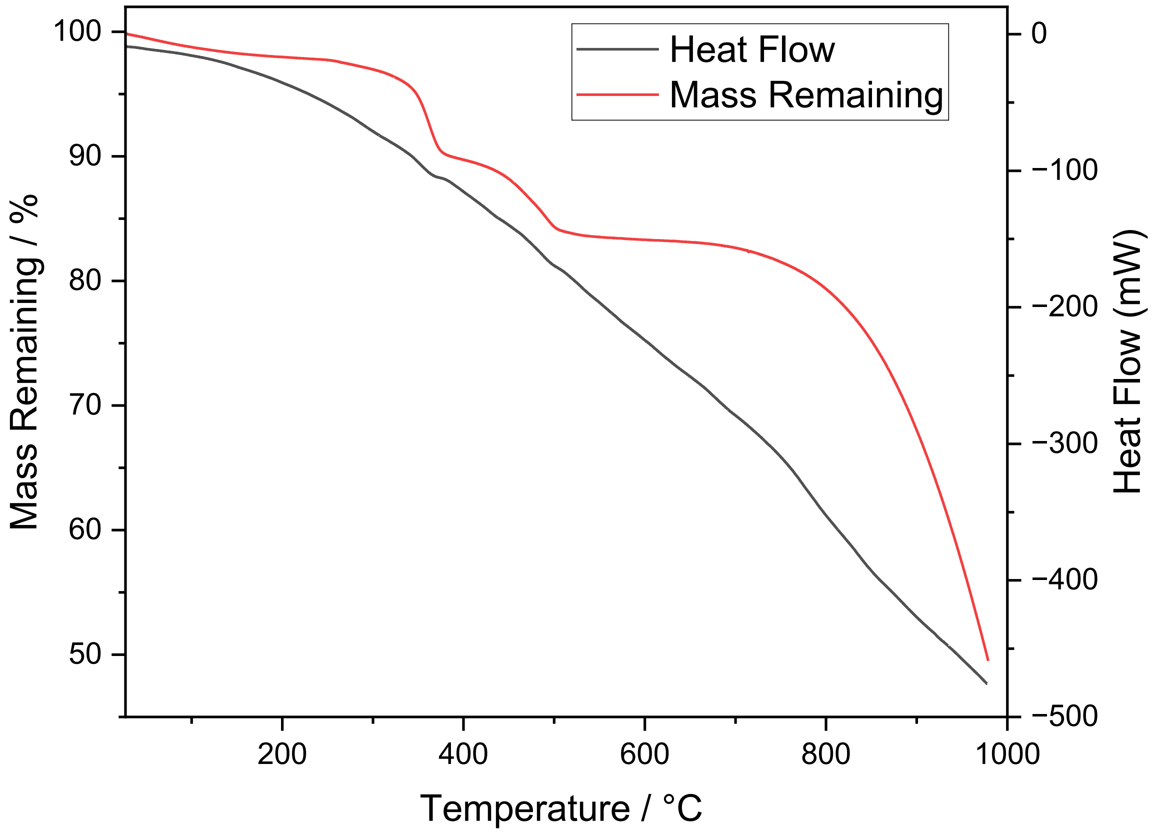

Figure S8: TGA-DSC of $SrRuO_3(OH)_2$

Table S6: Assignment of TGA of of $SrRuO_3(OH)_2$

**Temperature range**	**Decomposition**	**Expected Mass Loss (%)**	**Measured Mass Loss (%)**
< 300 °C	$SrRuO_3(OH)_2{\cdot}0.44H_2O \rightarrow SrRuO_3(OH)_2$ (loss of surface water)	3.0	3.0
300 °C – 400 °C	$SrRuO_3(OH)_2 \rightarrow SrRuO_4$	7.3	6.9
400 °C – 600 °C	$SrRuO_4 \rightarrow SrRuO_3$	6.1	6.1

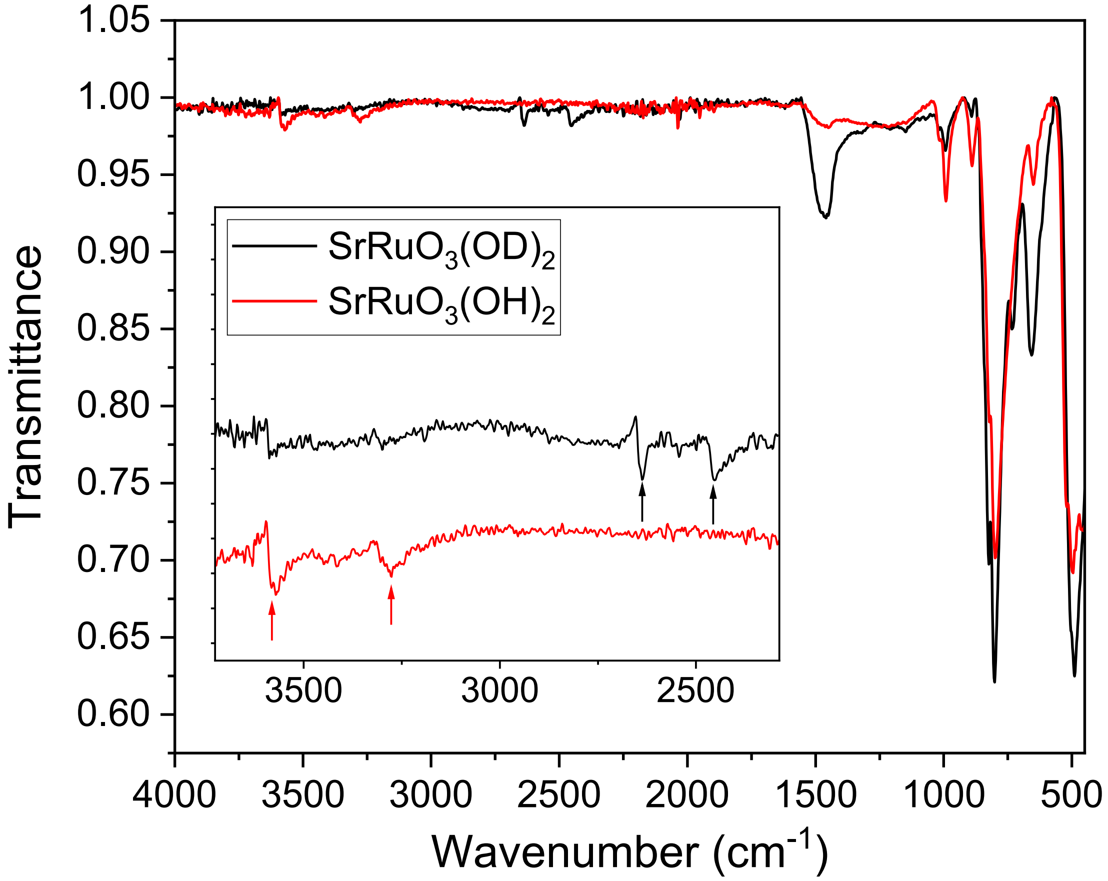

Figure S9: IR spectra of $SrRuO_3(OH)_2$ prepared in H_2O and D_2O. The inset shows the OH/OD stretch region, with the expected shift of bands indicated by arrows to lower wavenumber upon deuteration.

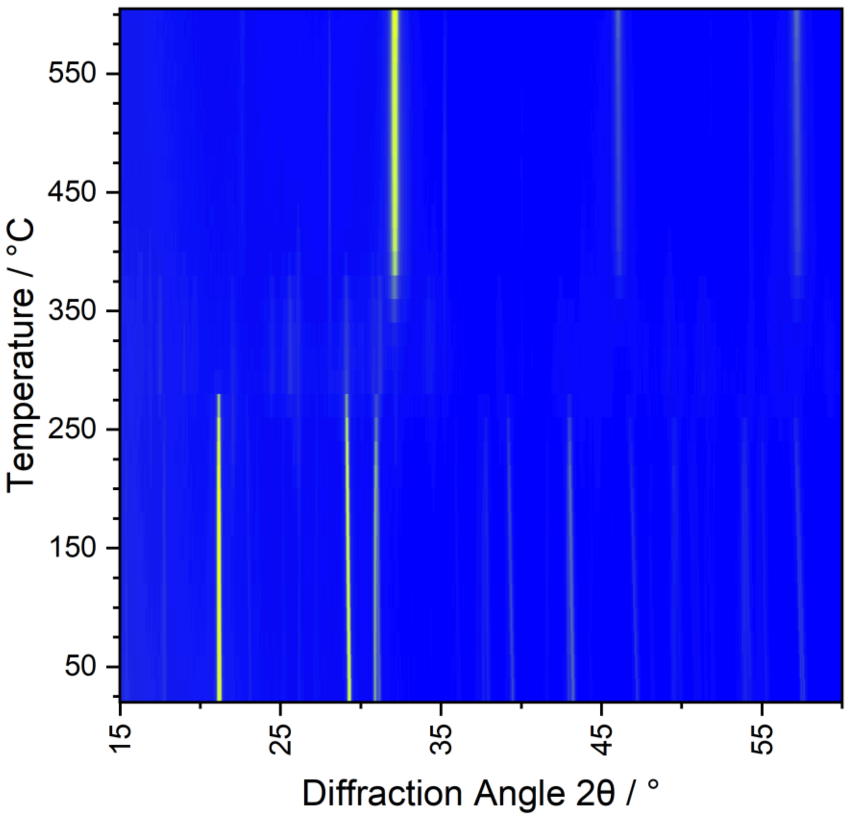

Figure S10: X-ray Thermodiffractometry of $SrRuO_3(OH)_2$ in air showing collapse above ~260 °C.

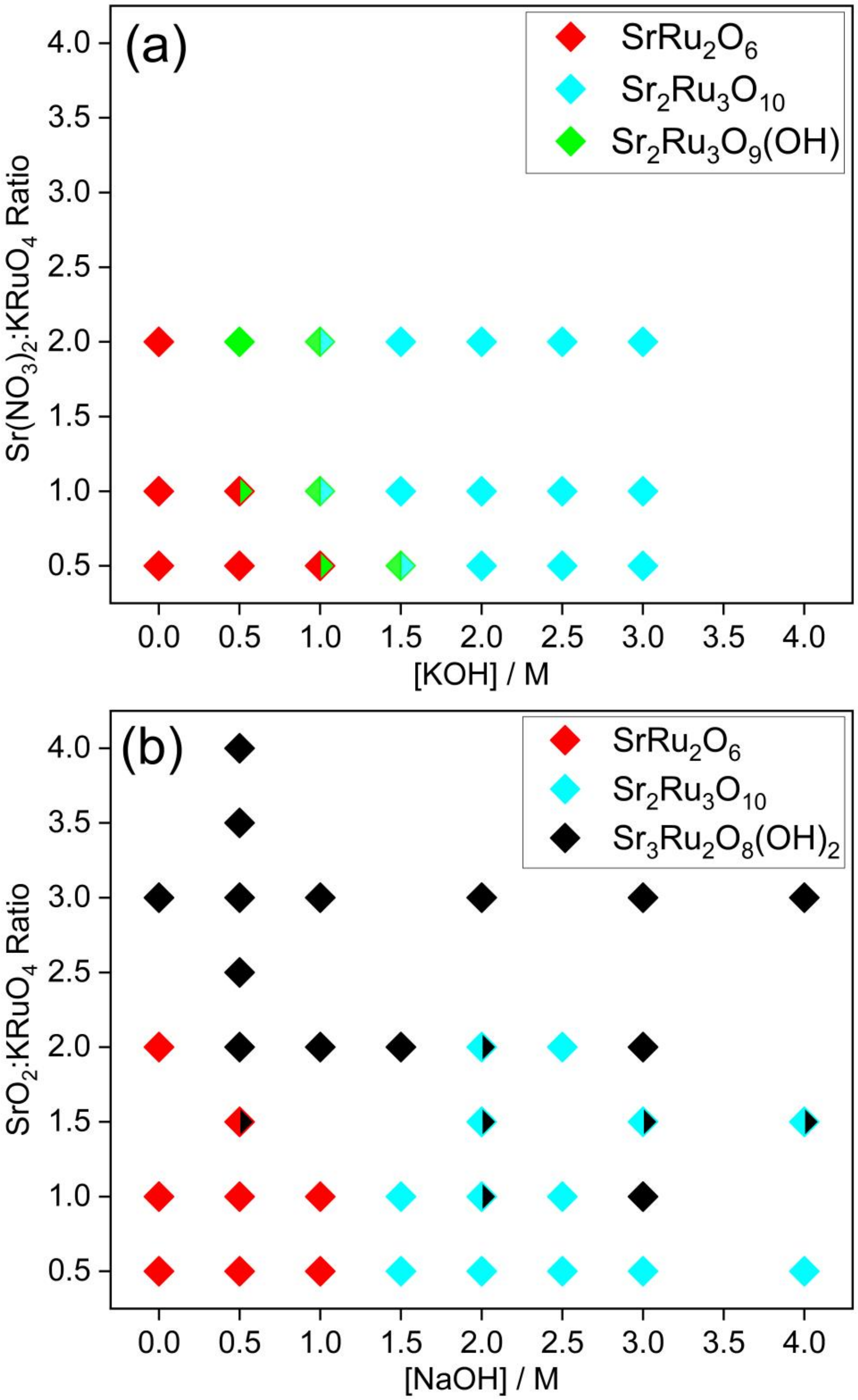

Figure S11: 'Synthesis maps' of strontium ruthenates under hydrothermal conditions (200 ºC). (a) represents the work of Marchandier *et al.* with results taken from [13] and (b) shows our work with the identification of a new phase labelled 'unknown', corresponding to $Sr_3Ru_3O_8(OH)_2$ identified in the present work.

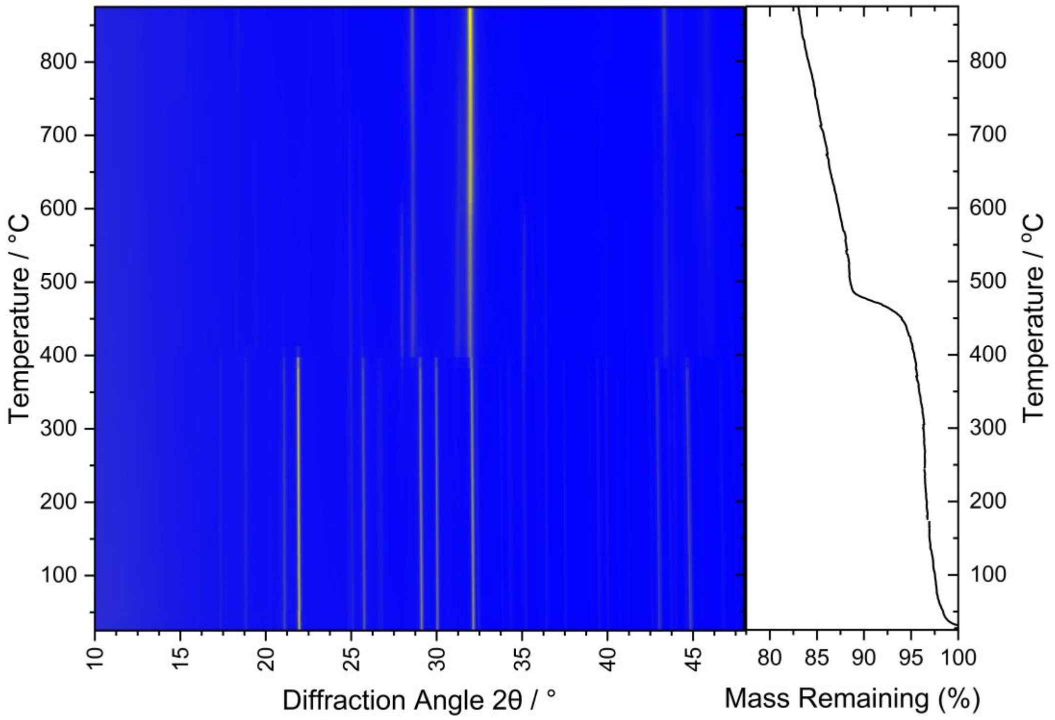

Figure S12: X-ray thermodiffractometry in air (left) and corresponding TGA (right) of $Sr_3Ru_2O_8(OH)_2$

Table S7: Assignment of TGA of $Sr_3Ru_2O_8(OH)_2$

**Temperature range**	**Decomposition**	**Expected Mass Loss (%)**	**Measured Mass Loss (%)**
< 300 °C	4 $[Sr_3Ru_2O_8(OH)_2 \cdot 1.35H_2O] \rightarrow$ 4 $[Sr_3Ru_2O_8(OH)_2]$ (loss of surface water)	3.70	3.74
430 - 510 °C	4 $[Sr_3Ru_2O_8(OH)_2]$ $\rightarrow 3[Sr_4Ru_2O_9] + 2[RuO_2] + 4H_2O + 5/2\ O_2$	5.83	6.15

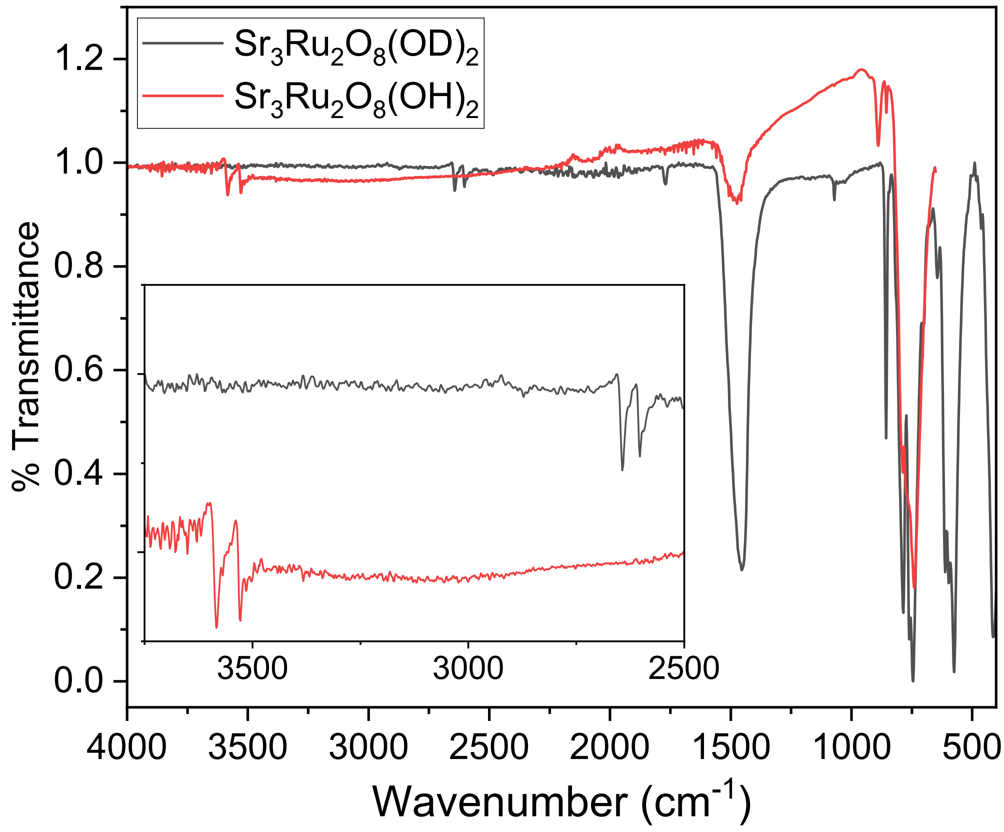

Figure S13: IR spectra of $Sr_3Ru_3O_8(OH)_2$ prepared in H_2O and D_2O. The inset shows the OH/OD stretch region, with the expected shift of bands to lower wavenumber upon deuteration

References

1. Popova, T. L.; Kisel', N. G.; Karlov, V. P.; Krivobok, V. I., Bivalent Metal Ruthenates (VI). *Russ. J. Inorg. Chem (Engl. Trans.)* **1981,** *26*, 1613–1615.
2. Nowogrocki, G.; Abraham, F.; Tréhoux, J.; Thomas, D., Configuration de l'ion ruthénate: structure cristalline du dihydroxotrioxoruthénate(VI) de baryum, $Ba[RuO_3(OH)_2]$. *Acta Crystallogr. B* **1976,** *32*, 2413-2419.
3. Fischer, D.; Hoppe, R., Zur Konstitution von Alkaliruthenaten (VI). 2. Über den Aufbau von $K_2[RuO_3(OH)_2]$. *Z. Anorg. Allg. Chem.* **1991,** *601*, 41-46.
4. Elout, M. O.; Haije, W. G.; Maaskant, W. J. A., Crystal structure determination of dipotassium dihydroxotrioxoruthenate(VI): configuration of the ruthenate ion and its electronic properties. *Inorg. Chem.* **1988,** *27*, 610-614.
5. Mogare, K. M.; Sheptyakov, D. V.; Bircher, R.; Güdel, H. U.; Jansen, M., Neutron diffraction study of the magnetic structure of Na_2RuO_4. *Eur. Phys. J. B* **2006,** *52*, 371-376.

6. Shikano, M.; Kremer, R. K.; Ahrens, M.; Koo, H. J.; Whangbo, M. H.; Darriet, J., Synthesis and Characterization of a Magnetic Semiconductor Na_2RuO_4 Containing One-Dimensional Chains of Ru^{6+}. *Inorg. Chem.* **2004,** *43*, 5-7.
7. Hansen, T.; Le Bail, A.; Laligant, Y., Synthesis and Structure Approach of Barium-Oxomercurato(II)-Oxoruthenate(VI) $BaHgRuO_5$. *J. Solid State Chem.* **1995,** *120*, 223-230.
8. Fischer, D.; Hoppe, R., Zur Konstitution von Oxoruthenaten(VI) 1. Über den Aufbau von $Cs_2[RuO_4]$. *Z. Anorg. Allg. Chem.* **1990,** *591*, 87-94.
9. Fischer, D.; Hoppe, R.; Mogare, K. M.; Jansen, M., Syntheses, Crystal Structures and Magnetic Properties of Rb_2RuO_4 and K_2RuO_4. *Z. Naturforsch. B* **2005,** *60*, 1113-1117.
10. Mogare, K. M.; Klein, W.; Peters, E.-M.; Jansen, M., $K_3Na(RuO_4)_2$ and $Rb_3Na(RuO_4)_2$, two new ruthenates with glaserite structure. *Solid State Sci.* **2006,** *8*, 500-507.
11. Fischer, D.; Hoppe, R., Über „Gemischt-Koordinierte" einkernige Anionen. 2. Ein Oxoruthenat(VI) neuen Typs: $CsK_5Ru_2O_9 = CsK_5[RuO_5][RuO_4]$. *Z. Anorg. Allg. Chem.* **1992,** *617*, 37-44.
12. Hansen, T., Copper(II)-Hydroxo-Oxoruthenate(VI): $CuRuO_2(OH)_4$ − Ab Initio Structure Determination by X-Ray Powder Diffraction. *Mater. Sci. For.* **1996,** *228-231*, 723 - 728.
13. Marchandier, T.; Jacquet, Q.; Rousse, G.; Baptiste, B.; Abakumov, A. M.; Tarascon, J. M., Expanding the rich crystal chemistry of ruthenium (V) oxides via the discovery of $BaRu_2O_6$, $Ba_5Ru_4O_{15}$, $Ba_2Ru_3O_{10}$ and $Sr_2Ru_3O_9(OH)$ by pH controlled hydrothermal synthesis. *Chem. Mater.* **2019,** *31*, 6295-6305.
